# Patients’ Experiences of Weight Regain After Bariatric Surgery

**DOI:** 10.1007/s11695-022-05908-1

**Published:** 2022-01-21

**Authors:** Liisa Tolvanen, Anne Christenson, Pamela J. Surkan, Ylva Trolle Lagerros

**Affiliations:** 1 Clinical Epidemiology Division, Department of Medicine Solna, Karolinska Institutet, Maria Aspmans gata 30A, 171 64 Stockholm, Sweden; 2 Center for Obesity, Academic Specialist Center, Stockholm, Sweden; 3 Department of International Health, Johns Hopkins Bloomberg School of Public Health, Baltimore, MD USA

**Keywords:** Bariatric surgery, Body weight trajectory, Interviews, Obesity, Thematic analysis

## Abstract

**Purpose:**

Bariatric surgery is a successful obesity treatment; however, an estimated 1/5 of patients have regained more than 15% of their body weight 5 years post-surgery. To increase the understanding of patients who experienced weight regain after bariatric surgery, we conducted a qualitative study.

**Materials and Methods:**

We recruited 16 adult participants (4 men, 12 women) at an obesity clinic in Stockholm, Sweden, 2018 to 2019, and performed semi-structured individual interviews. The transcribed recorded interview data was analyzed with thematic analysis.

**Results:**

Participants had undergone gastric bypass surgery on average 10 years prior to study and regained 36% (range 12 to 71%) of their weight from their nadir. Participants experienced challenges such as eating in social settings, loneliness, family difficulties, increases in appetite, and physical and mental health problems, which distracted them from weight management. Participants responded to weight regain with emotional distress, particularly with hopelessness, discouragement, shame, and frustration (theme: loss of control and focus). Nonetheless, participants experienced remaining benefits from the surgery, despite weight regain. Social support, self-care, and behavioral strategies were perceived as facilitators for weight management (theme: reducing the burden of weight management).

**Conclusions:**

Weight regain after bariatric surgery was perceived to be an unexpected and difficult experience that induced hopelessness, discouragement, shame, and frustration. Results indicate that internal and external circumstances such as psychosocial factors, changes in appetite, and physical and mental health problems may contribute to loss of control over weight. Social support, self-care, and behavioral strategies might facilitate long-term post-surgical weight management.

**Graphical abstract:**

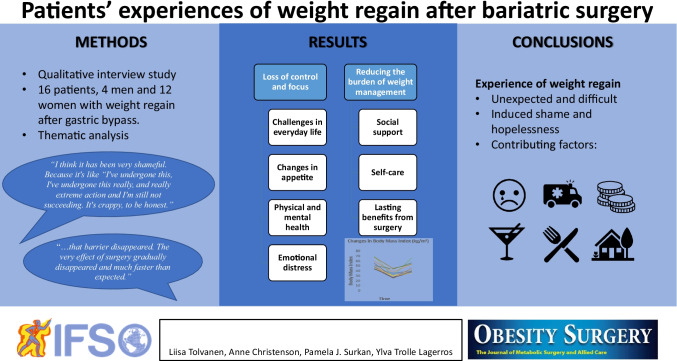

**Supplementary Information:**

The online version contains supplementary material available at 10.1007/s11695-022-05908-1.

## Introduction

Compared to lifestyle interventions, bariatric surgery is superior for long-term weight loss maintenance and improvement in overall health and quality of life [1, 2]. However, weight loss after bariatric surgery varies between individuals [3]. Most patients regain some weight after their lowest weight has been reached [4]. Studies report large differences in the prevalence of weight regain in the bariatric population. The Longitudinal Assessment of Bariatric Surgery study (LABS) shows mean weight regain of about 4% after Roux-en-Y gastric bypass (RYGB) 3 to 7 years after surgery [5], while other studies e.g. from Sweden [6] and the Netherlands [4] report that 20 to 24% of patients have regained more than 15% of their body weight 5 years after RYGB or sleeve gastrectomy. The exact prevalence of weight regain is difficult to estimate, since a definition for weight regain has not been established [4, 7].

Genetic factors [8], increases in gut hormones such as glucagon-like-peptide-1 (GLP-1) and peptide YY (PYY) [9], and decreases in the production of ghrelin play an important role in appetite regulation and post-surgical weight outcomes [10]. Anatomical failures related to surgical procedures, such as gastro-gastric fistula or dilated gastric pouch, may contribute to weight regain [7]. Post-bariatric hypoglycemia has also been associated with weight regain [11]. Additionally, unhealthy eating habits and sedentary lifestyle [12], poor mental health, substance use, eating disorders [13, 14], and the lack of nutritional follow-up may promote weight regain after bariatric surgery [12].

A qualitative study demonstrates that the struggle with weight management continues for some patients, while others have long-term beneficial effects of their surgery even 10 years later [15]. Some patients fear weight regain [15–17] and those who experience weight regain often perceive it as a personal setback [17, 18].

To better support these patients, we need to understand how patients experience weight regain after bariatric surgery and what factors they believe complicate weight management. Since weight regain after bariatric surgery is a complex phenomenon, qualitative research on patient perceptions may enable increased understanding of barriers and facilitators during weight regain.

The purpose of this study was to increase the understanding of the patient experience of weight regain after bariatric surgery and factors that may improve post-bariatric care.

## Materials

### Participants and Settings

Participants were recruited by purposive sampling [19] among treatment-seeking patients at an obesity clinic in Stockholm, Sweden, between April 2018 and December 2019. All participants had been referred for medical obesity treatment by their primary care physician or obesity surgeon. The medical staff at the obesity clinic invited eligible patients face-to-face to participate in the study.

Eligible participants had a body mass index (BMI) ≥ 35 kg/m^2^, were 18 years and older, and had a weight regain ≥ 10% after sleeve gastrectomy or gastric bypass. Patients with ongoing treatment contact with the first author (LT) were excluded. Oral and written study information was provided. Participation was voluntary and there were no incentives to encourage participation. Three women declined participation due to the lack of time. They had similar BMI and weight regain as included participants but were marginally younger (mean = 37 years), compared to 49 years among the participants.

## Methods

### Study Design

**Fig. 1:**
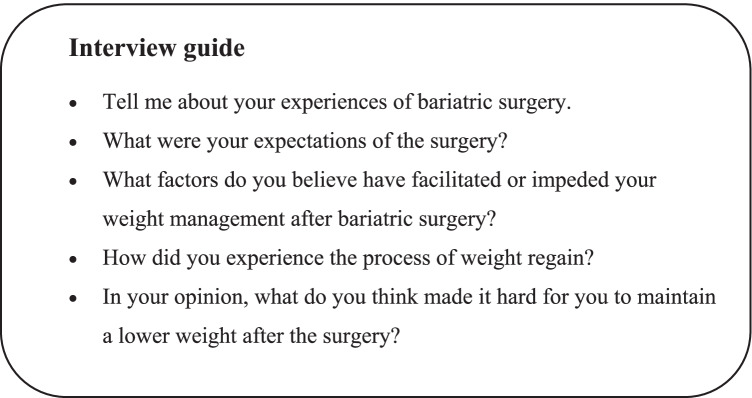
The interview guide used in the study

We chose a qualitative inductive approach with in-depth interviews as it enabled us to explore participants’ verbal expressions of lived experiences [20]. A pilot-tested semi-structured interview guide (Fig. 1) [19] with open-ended questions about experiences of weight regain was used, including background questions regarding age, health status, type of surgery, weight data, occupation, and close relationships. Although participants had the option of choosing the location of the interview, all interviews were conducted at the obesity clinic. Participants were interviewed once, and interviews lasted for 32–79 min (mean 60 min). Only the participant and the first author were present. Notes were taken during the interview and interviews were recorded digitally and transcribed verbatim by the first author.

### Data Analysis

Thematic analysis by Braun and Clarke [20] was adapted and used to analyze the transcribed interviews. Phase 1: We read through the entire data set repeatedly, noting primary ideas. Phase 2: We identified data extracts corresponding with the study questions and coded them with initial labels. Phase 3: We collated and sorted codes into preliminary themes (Supplemental, Table 1S). A thematic map was used to compare codes with each other and to find relationships within themes. Phase 4: We refined and reviewed the themes with attention to internal coherence for coded data extracts. Phase 5: We established the final themes and organized extracted data under each of them. The final results were checked against the data set and structured by sub-themes to emphasize the essence within each theme. Phase 6: Quotes were chosen to demonstrate the authenticity of the analysis.

### Reflexivity and Trustworthiness

To reduce the impact of any preconceptions of the first author, we performed analyst triangulation [19] where the first author (she is a PhD-student, MSc in public health, dietitian specialized in obesity and cognitive behavioral therapist) and the second author (AC) (she has a PhD, is a physiotherapist and cognitive therapist specialized in obesity) separately coded two interview texts to detect any discrepancies in how to interpret the data. The first author and the second author collaborated during phases 2 and 3 in developing the preliminary themes. During phases 2 and 3, the first author manually collated and coded data extracts and sorted them into preliminary themes. During phases 4 and 5, all authors collaborated in refining and reviewing themes until agreement was reached. The second to the last author (PJS), is a female experienced qualitative researcher, ScD, and professor in global health. The last author (YTL) is a female MD specialized in internal medicine/obesity, PhD, MPH, and associate professor. All authors contributed critical feedback, and final themes evolved in collaboration between the authors. The main themes were checked against the data extracts as well as against the preliminary themes to maintain consistency. After thirteen interviews, a joint decision was made that three more interviews would be needed to achieve sufficient variation in the sample and to reach informational redundancy [19]. Participants were invited to check the transcribed text to ensure correctness. No changes were suggested.

We have used the consolidated criteria for reporting qualitative research (COREQ) [21].

## Results

### Participants

**Fig. 2:**
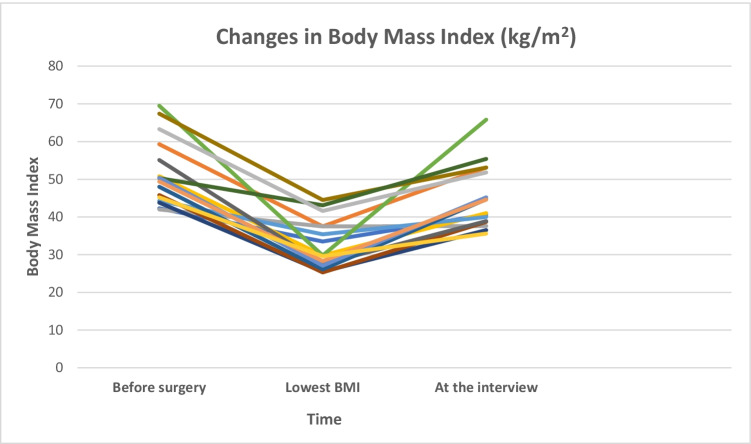
Individual changes in body mass index after gastric bypass among participants (*n* = 16)

Sixteen (*n* = 16) adult participants (4 men, 12 women) with weight regain after bariatric surgery participated. The mean BMI was 46, with on average a 36% weight regain from their nadir. According to most participants (*n* = 13), their weight regain started between 1 and 5 years (mean 2.6 years) after surgery. Some (*n* = 3) could not remember when weight regain started. Table 1 displays participant characteristics and Fig. 2 shows changes in BMI trajectories from time of bariatric surgery to the interview. All participants underwent gastric bypass between 2004 and 2016. Three participants had undergone previous bariatric surgeries (sleeve gastrectomy *n* = 1, gastric banding *n* = 2), but due to complications of the first procedure, gastric bypass was performed at a later stage. Participants differed regarding occupational status, marital status, and ethnicity; 31% (*n* = 5) originated from outside Europe.

**Table 1:** Participant characteristics (*n* = 16)

Characteristics	
Female, *n* (%)	12 (75)
Age, in years, mean (range)	49 (20–64)
Marital status, *n* (%)	
Married/partner	12 (75)
Unmarried/single/divorced	4 (25)
Occupation, *n* (%)	
Employee/self-employee/student	13 (81)
Income support	3 (19)
Origin, *n* (%)	
Nordic	11 (69)
Middle East, Asia, South America	5 (31)
Psychiatric comorbidities, *n* (%)^a^	10 (63)
Type of surgery^b^	
Roux-en-Y gastric bypass (RYGB), *n* (%)	16 (100)
Time from surgery in years, mean (range)	10 (3–15)
Total weight loss from surgery, %, mean (range)	35 (14–50)
Total weight regain from nadir, %, mean (range)	36 (12–71)
Body mass index (BMI) (kg/m^2^)	
Pre-surgery	52 (42–70)
Lowest weight	34 (25–49)
After weight regain	46 (36–66)

^a^Depression, anxiety, self-harm, eating disorder, and/or neuropsychiatric condition (self-reported)

^b^RYGB was a re-operation for three participants (previous surgery: sleeve gastrectomy *n* = 1, gastric banding *n* = 2)

### Themes

**Fig. 3:**
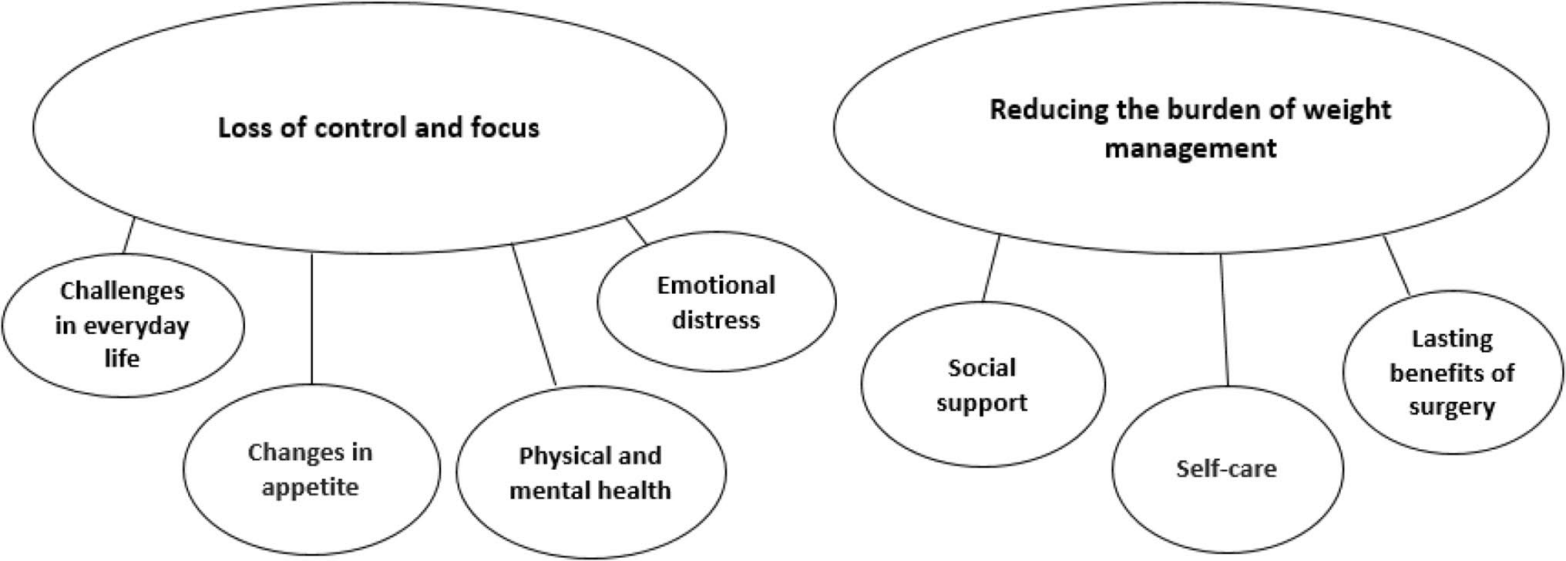
Final thematic map demonstrating two major themes and seven sub-themes

Two major themes were found (Fig. 3). (1) *Loss of control and focus* and (2) *reducing the burden of weight management*. Seven sub-themes illustrated participant experiences during post-surgery weight regain.

#### Theme 1: Loss of Control and Focus

Most participants perceived the process of weight regain as a slow and accompanied gradual loss of control and focus. Participants were unprepared for weight regain and had expected to maintain a lower long-term weight. Participants perceived weight regain as stressful, shameful, and frustrating. They expressed hopelessness and discouragement. Meanwhile, support from healthcare professionals and family members was perceived as scarce [22].

#### Challenges in Everyday Life

Work-related stress, financial concerns, unemployment, unstable housing situations, pregnancies, prioritization of the well-being of family members, and family conflicts were examples of situations that compromised weight control.

We have had a lot of trouble with my stepson, a lot of conflicts. He has not been well, we have not felt well. It has affected the whole family situation. And I can say, that’s made my weight increase. (Interview 15)

The home food environment commonly remained unchanged post-surgically. As the effects of surgery declined with time, it became harder to eat small portions and avoid unhealthy food. Initially, friends and family members actively supported healthy food choices, but with time they relapsed into old habits. Participants challenged the effects of the surgery by eating foods high in sugar and fat, sometimes encouraged by others.

And then he ordered a pizza slice or whatever it was, and then I said “Are you able to eat that? I´m not.” Then he said “Oh, you are going to, and now I’ll show you how to drink a beer”. So, then I tried too. It’s very sad that I got that lesson. (Interview 13)

#### Changes in Appetite

Participants described that bariatric surgery initially changed their perceptions of hunger, and satiety, and provided a sense of control. However, eventually portion sizes increased and hunger returned, and for some also cravings for sweets. Participants had trusted the procedure to prevent them from eating too much, or unhealthy, and from regaining weight. Instead, the effects of surgery eventually disappeared.

…that barrier disappeared. The very effect of surgery gradually disappeared and much faster than expected. (Interview 2)

Some participants feared that increased hunger implied complications or abnormal changes in the bowel or gastric pouch. Those who had been examined by a surgeon felt relieved that everything looked fine. Meanwhile, other participants mentioned even forgetting that they had undergone surgery. Yet others described post-surgical symptoms such as nausea, diarrhea, dizziness, fatigue, or cramping, and used snacking or bedrest to cope.

#### Physical and Mental Health

As several participants were affected by acute and chronical diseases, weight management became less central and healthcare visits focused on other conditions.

If I would not have had a kidney transplantation, I think I may still have been quite weight stable. …but it [the kidney transplant] had been crucial. It’s about life and death. Then like, the weight is a bit less important actually. … I think I might have denied it. Ok. I regained a bit, but there’s something much more important to care about—kidney function and such… (Interview 9)

Participants also experienced mental health issues. Emotional eating served as a coping mechanism for mental health issues.

Then [when feeling lonely] comfort eating starts again, because you think that, it’s just no use. ‘I can eat my chips, I can eat my chocolate, drink my soda’. (Interview 1)

Some participants mentioned that the use of medications such as cortisone and antidepressants may have contributed to weight regain.

I think it [weight regain] started with, it started with me getting cortisone and then I gained 10 kg very fast. … I have ended up in a vicious cycle, that I gain 2 to 3 kg each year. (Interview 7)

Most were aware of the risk of alcohol addiction. However, some participants described hazardous alcohol drinking during weight regain.

Then a lot of parties. Lots of alcohol. Excessively too much. … So, as I said, alcohol has made me regain weight. (Interview 16)

A couple of years ago, I never wanted to call myself an alcoholic, but I drank an awful lot of alcohol to numb myself. It was mostly numbing me. I felt so bad, so I just wanted to sleep so I drank alcohol. But now I have not been drinking for several years. (Interview 2)

#### Emotional Distress

Both losing and regaining weight were experienced stressful and had a negative impact on body image. Weight regain was unexpected and participants were not prepared for it. They expected to lose a considerable amount of weight and keep it of permanently.

I did not believe I could regain the weight. I actually did not believe that… (Interview 4)

I trusted that I would lose a lot more weight than I did. I lost about 25 kg. I had hoped, in my wildest dreams, that I might be able to lose 50 kg. And that I could keep the weight off. (Interview 5)

Participants feared regaining all the weight they lost. They experienced weight regain as devastating and expressed hopelessness and discouragement. They were frustrated about difficulties in losing weight and keeping it off. Weight regain contributed to feelings of shame, guilt, and despair. Self-blame was common.

I think it has been very shameful. Because it’s like “I’ve undergone this, I’ve undergone this really, and really extreme action and I’m still not succeeding. It’s crappy, to be honest.” (Interview 15)

…this has affected my entire quality of life. I feel like half a human being. Not happy at all. And I always try to do as I should [cries], and I search for mistakes…I eat too little. (Interview 12)

Some participants worried about their future health and quality of life. They expressed frustration as they tried to be strict in their dieting and exercise, but with disappointing weight loss results.

It’s my biggest feeling that I’m frustrated, that nothing happens and even if I eat right and exercise right, nothing happens. My weight is completely stable. (Interview 7)

Comments from others further contributed to feelings of shame and guilt.

She’s skinny, my little sister is skinny and I’m chubby. My dad has always had opinions. ‘No, but you should be like her, you should be skinny and so on’. So it has been hard there too. I have always struggled to be just as slim, but I have not succeeded. (Interview 4)

#### Theme 2: Reducing the Burden of Weight Management

##### Social Support

Most participants emphasized the importance of having someone who was able to give support in a way that participants felt understood.

Today, I have support and can talk about my problems with a contact person [a person from social services]. It is a luxury, I have to say. I’m just now actually starting to live. (Interview 3)

Participants requested more psychological support and evidence-based and concise recommendations about diet from healthcare professionals, as well as empathic and individualized support.

I need to hear what I am doing right, but also how I can do more right. (Interview 5)

##### Self-care

Participants had gained insights about the difficult task of changing lifestyle and eating habits permanently.

I tried different diets again. I started with the GI [Glycemic Index] method. But then you relapse. I lost weight with it, but it’s really about changing your way of thinking completely. And it does not really work that way. (Interview 14)

They stated that it was time to prioritize their own needs and claimed they would do anything to lose weight. Several participants requested a re-surgery.

…if I could get a new operation to get a fresh start. Because now I am a little more mature than before and research and care has improved, you could get more support. It would be perfect, then I would be so happy to get a second chance. (Interview 11)

Some participants used strategies to reduce alcohol intake, such as changing their social life or getting support from a specific family member. They described behavioral strategies that could be supportive in weight management such as eating slowly and having a plan for meals, portion sizes, and sugar intake.

So keep your meal times, it’s very important. Because it is the same thing there, no one told me that ‘you should eat between 5 to 6 times / day’ that is what I have heard. Now I think you should eat up to 6–7 times a day even. So, I have a “food-and-sleep-alarm’ that goes off and reminds me when to eat, because otherwise I forget it.” (Interview 7)

Participants had gotten insight into the importance of regular self-weighing. Some had recently started to become more physically active. Those participants emphasized the physical and mental positive effects of exercise.

Today I have been at the gym for the first time, which is why I have so much packing here. (Laughter). Today, I exercised for the first time in 10 years or more. So today is a big day for me. (Interview 9)

##### Lasting Benefits of Surgery

Despite setbacks and disappointments, except for one person, no one regretted having surgery, and most participants weighed less than before surgery.

It [surgery] has saved my life. I felt so bad and could barely move. So there is no doubt that it is an effective tool. (Interview 15)

Participants had noticed that when they ate healthier it became easier to limit the amount of food, as if some of the effects of surgery were still there.

I’ve noticed one thing. When I start a diet or try to lose weight, if I keep it up for a couple of days, it feels like I’ve recently undergone the surgery. I have tried several times and it feels for sure that now it’s back, the small stomach. (Interview 10)

## Discussion

In this qualitative study of experiences of weight regain after bariatric surgery, participants perceived a loss of control and of focus on weight management during weight regain. They were affected by internal and external circumstances such as psychosocial factors, increased appetite, and physical and mental problems. Participants were unprepared for weight regain and reacted with emotional distress, i.e., hopelessness, discouragement, shame, and frustration. Regaining weight was a devastating experience that contributed to a negative spiral in weight management. Still, participants felt that social support, self-care, and behavioral strategies could facilitate weight management.

Participants had expected surgery to provide long-term control of their eating habits and weight. Patients expect bariatric surgery to end the struggle with weight and eating [23, 24]. In this study, those initial feelings of confidence and improvements in eating behaviors were replaced by loss of control. Participants speculated that loss of control was caused by not adhering to dietary recommendations or due to anatomical reasons. However, they described increased hunger and decreased satiety with time, which may indicate hormonal and metabolic changes affecting appetite regulation [9, 25].

The emotional distress that participants perceived during weight regain was fueled by the experience of “a double failure” [26], initially an inability to maintain a normal weight and subsequently a failure to achieve a successful post-surgical result. The shame participants experienced, related to societal weight bias, may have contributed to non-functional coping strategies, such as emotional eating, grazing, or restricted eating that are in line with studies reporting associations between weight regain and maladaptive eating behaviors [27, 28]. Negative self-image, maladaptive eating behaviors, substance use, and overall impaired psychosocial functioning have in turn been associated with internalized weight bias and further weight management difficulties [29, 30]. Though symptoms of binge eating or depression seem to decrease after surgery [31, 32], our study indicated difficulties with eating behavior and mental health problems as drivers of weight regain.

Some participants reported harmful or hazardous alcohol consumption, which complicated their weight management. There is an increased risk of alcohol use after gastric bypass surgery, with about one-fifth of those receiving surgery reporting symptoms of alcohol use disorder 5 years after surgery [33]. Alcohol absorption and its metabolism is modified by anatomical changes following gastric bypass surgery [34]. Post-operative alcohol use has been identified as a predictor for weight regain [27]. Furthermore, psychiatric comorbidities, like prior depression, have been reported to be high among patients with hazardous alcohol consumption after gastric bypass surgery [35]. The present study population mentioned psychiatric comorbidities, as well as altered effects of alcohol, as drivers of increased alcohol use. Further, it has been suggested that alcohol could be used to regulate emotions [14]. Theories about “addiction transfer” have been proposed but they are controversial [36, 37]. Addiction transfer refers to a shift, where food rewards are replaced with other substances post-surgically, which may also contribute to weight regain.

As patients may forget pre-operative information [38], the risk of weight regain is a message that may need to be repeated along with encouragement to seek help if needed. To counteract weight regain, healthcare professionals need to identify patients who are at risk of or are already regaining weight [7], and offer individualized treatment with an empathetic approach [39]. Multidisciplinary care and follow-up should include components that support long-term mental and physical health [40], as desired by participants in the present study. Besides medical nutrition therapy [41], psychosocial [42] and behavioral interventions [43] are important cornerstones in supporting lifestyle changes and self-care in patients with weight regain. Treatment with anti-obesity medications may facilitate weight management and weight loss in patients with weight regain in the same way it does for patients who have not undergone surgery [44–46]. Further, revisional surgery may be a treatment option for some patients [46, 47].

Participants in our study had a pre-surgical mean BMI of 52 kg/m^2^. Patients with a pre-surgical BMI of ≥ 50 kg/m^2^ are more likely to regain weight, while a lower pre-surgical BMI has been associated with more successful weight loss outcomes 12 to 36 months after surgery [48]. These findings and the fact that our sample had a mean BMI > 50 kg/m^2^ indicate that patients in this BMI range may need even more intensive post-bariatric support.

Our participants had undergone surgery on average 10 years ago. A limitation was that all our participants had undergone gastric bypass surgery. This may affect the transferability of these results to patients with other bariatric procedures. Gastric bypass has historically been the main bariatric procedure in Sweden, and only recently sleeve gastrectomy became equally common [49]. Otherwise, participants reflected the current Swedish bariatric population; approximately a quarter of the patients undergoing bariatric surgery in Sweden are men and the average age is 41 years at the time of surgery [49]. Another limitation was that we only included treatment seekers. Non-treatment seekers may have provided different narratives.

Previous psychological or psychiatric conditions may be predictors of weight regain. Low self-esteem, for example, has been shown to be associated with weight regain after gastric bypass surgery [50]. Although we did not assess self-esteem, almost two-thirds of the participants self-reported psychiatric or neuropsychiatric comorbidities. A previous study has shown that psychological improvements after bariatric surgery may slowly decline with weight regain and with time from surgery [51], indicating the importance of including self-esteem and psychiatric comorbidities in future studies of weight regain.

Participants in the present study had undergone bariatric surgery 3 to 15 years (mean 10 years) prior to participation. For some participants, it was difficult to remember when the weight regain started. Procedures, perioperative protocols, and post-operative management for bariatric surgery evolve over time. The patients in our study had gone through bariatric surgery over a number of years and may have received different information about the risk for weight regain. A short interval since time to surgery could have led to information about patient experiences of a particular protocol. The present study includes findings from a wide range of BMI categories. The mean group BMI was slightly higher than most bariatric populations. In a qualitative perspective, this may be seen as a strength as it can provide more breadth and depth to the descriptions of the phenomenon of interest. It may also enable transferability to a larger range of pre-surgical BMI categories.

## Conclusion

Weight regain after bariatric surgery was perceived to be an unexpected and difficult experience that induced hopelessness, discouragement, shame, and frustration. Our results indicate that internal and external circumstances, e.g., changes in appetite and physical and mental health problems, may contribute to a loss of weight control. Social support, self-care, and behavioral strategies might facilitate long-term post-surgical weight management. Findings from this qualitative study may be hypothesis generating for future quantitative studies.

## Supplementary Information

Below is the link to the electronic supplementary material.

Supplementary file1 (DOCX 14 KB)

Supplementary file2 (PDF 545 KB)
