# Supplementary material for: Patients’ Experiences of Weight Regain After Bariatric Surgery
Source: Obes Surg. 2022 Jan 21;32(5):1498–507. doi: 10.1007/s11695-022-05908-1 (PMC8986695; doi:10.1007/s11695-022-05908-1)
Supplement: Supplementary file 1 — Supplementary file1 (DOCX 14 KB) [file 11695_2022_5908_MOESM1_ESM.docx]

**Supplemental**

Table 1S. Examples of early phase of the analysis process

| **Data extract** | **Coded for** | **Preliminary themes** |
| --- | --- | --- |
| “And then you begin to realize that it is still me who is ... Then you begin to feel "I am useless". You feel bad. Why can´t I control my food intake?” | Self-accusations and feelings of worthlessness | Struggle with negative  emotions |
| “Now I have decided, dammit, now I'm going to take care of myself. I'm not a coward, so I can say no. So I thought, now I'm going to fight for myself.  I have fought for other people all the time, but now I will take care of myself, not just others.” | Necessity to prioritize own needs | Insights about oneself |
